# Supplementary material for: Exploratory Gene Ontology Analysis with Interactive Visualization
Source: Sci Rep. 2019 May 24;9:7793. doi: 10.1038/s41598-019-42178-x (PMC6534545; doi:10.1038/s41598-019-42178-x)
Supplement: Supplementary file 1 — Supplementary Information [file 41598_2019_42178_MOESM1_ESM.pdf]

Exploratory Gene Ontology Analysis with Interactive Visualization
Supplementary Information

Junjie Zhu,[1,†] Qian Zhao,[2] Eugene Katsevich,[2] and Chiara Sabatti,[2,3,†]

[1] Department of Electrical Engineering, Stanford University, Stanford, CA, USA

[2] Department of Statistics, Stanford University, Stanford, CA, USA

[3] Department of Biomedical Data Science, Stanford University, Stanford, CA, USA

[†] To whom correspondence should be addressed: jjzhu@stanford.edu, sabatti@stanford.edu

Contents

Supplementary Figures 3

Supplementary Videos 9

0.1 Introduction to Focus and Context Graphs 9

0.2 Interactions to Explore the GO 9

0.3 Overview of Graph Layouts 9

0.4 Power Analysis Workflow 9

Supplementary Algorithms 10

A.1 The constrained GO DAG refinement algorithm 10

A.2 The bubble float algorithm 11

A.3 The focus graph node ordering algorithm 12

Supplementary Note 1: Theoretical Analysis of the Algorithms 13

1.1 Notation and definitions 13

1.2 Necessary and sufficient conditions for the buoyant layout 14

1.3 Analysis of the bubble float algorithm 15

1.4 Analysis of the graph refinement algorithm 15

Supplementary Note 2: Framework for testing for enrichment 17

2.1 Notation Summary 17

2.2 Self-contained and competitive hypotheses 18

2.3 Data Generation 18

2.4 Self-contained and Competitive testing procedures 19

2.5 Multiple Testing Correction 20

Supplementary Note 3: Case Studies 21

3.1 Visualization of enriched terms in the GWAS example 21

3.2 Visualization of enriched terms in the ChIP-seq study 21

3.3 Simulation of single-cell expression distinguished by cell cycle signatures 22

3.4 Power analysis for the heart development study 23

3.5 Comparison between DAGGER and BH 24

Supplementary References 25

Supplementary Figures

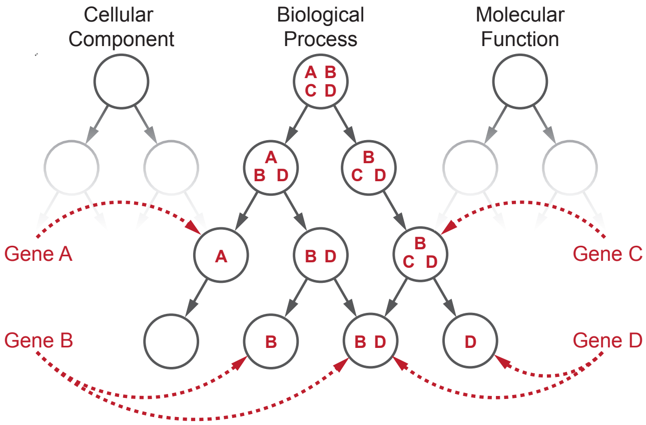

Supplementary Figure 1: DAG representation of the GO. The DAG structure of the GO. The GO can be represented as a graph where each node represents a GO term, and each directed link pointing from a parent to a child represents the semantic relationship with two terms. The child term can be related to the parent term via an "is a", "part of" or "regulates" relation. There are three root nodes: "biological process", "molecular function" and "cellular component", and each of their descendants are connected with directed edges in an acyclic fashion. Genes can be simultaneously annotated to different terms, and their annotations propagate upwards in the hierarchy, resulting in the gene sets in the nodes.

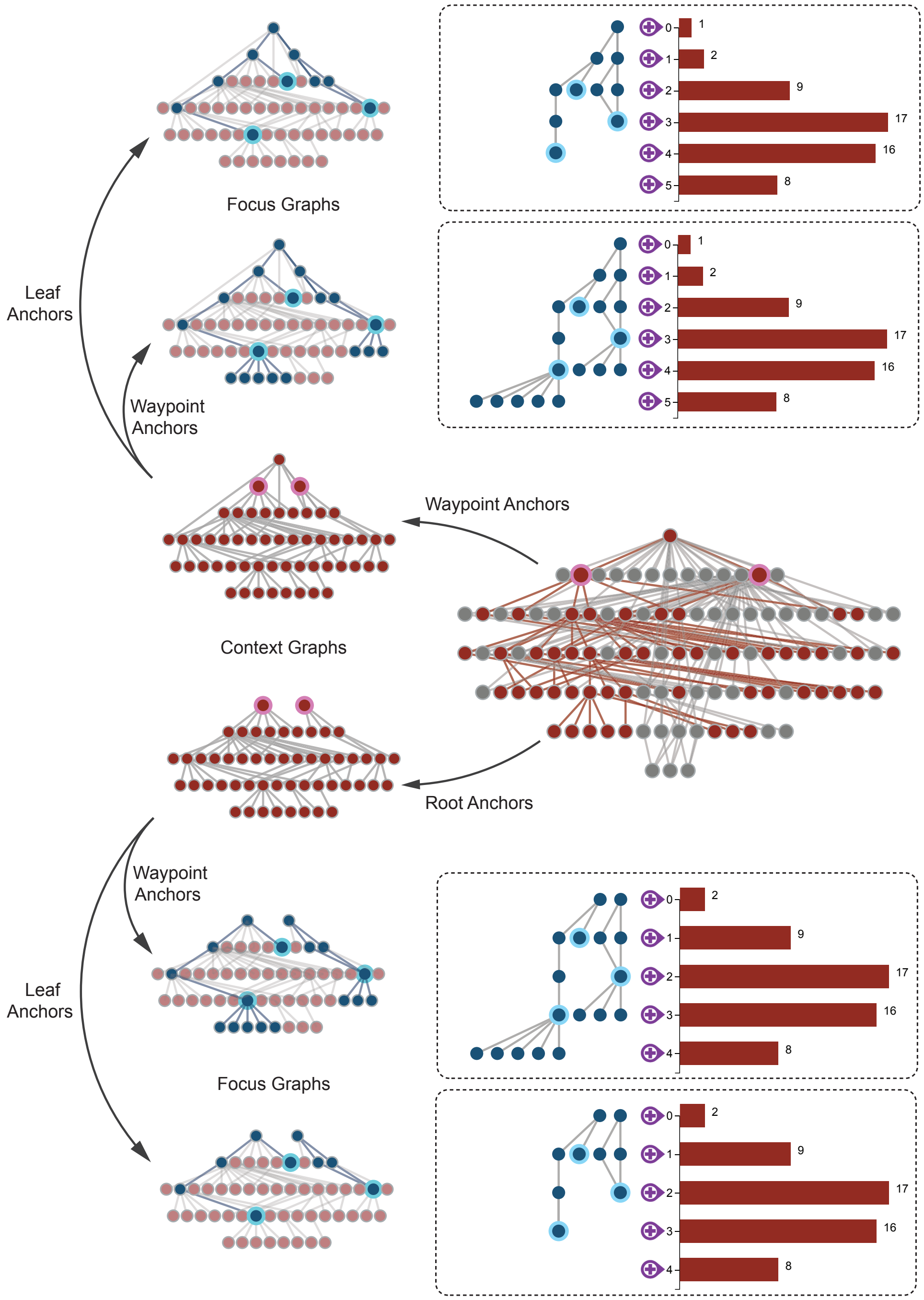

Supplementary Figure 2: Construction of focus and context graphs and their display. Context graphs (shown in red) can be defined w.r.t. a full DAG based on context anchors (with pink borders), and focus graphs (shown in blue) can be similarly specified w.r.t. the context graph using focus anchors (with aqua borders). Waypoint anchors include both ancestors and descendants, whereas root anchors include only descendants and leaf anchors include only ancestors. For the display of AEGIS, the focus graph (on the left of each sub-panel) is rendered with leveled nodes and their links, and the context graph (on the right of each sub-panel) is represented as a summary bar graph where each row is the number of nodes at the given level.

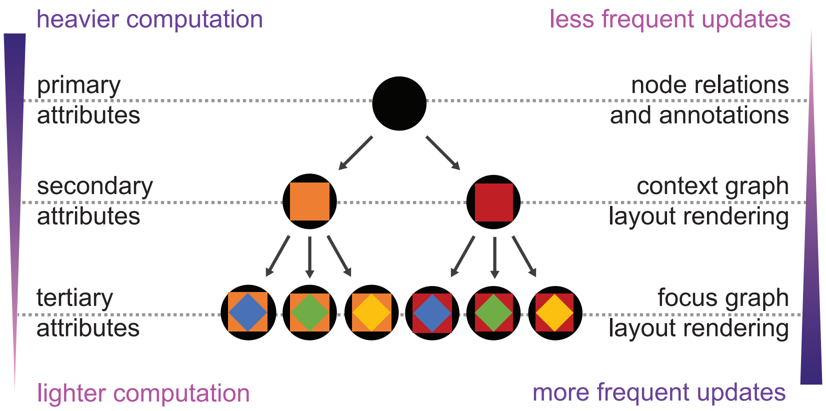

Supplementary Figure 3: Hierarchical data structures in AEGIS. Data objects in the software implementation of AEGIS consist of hierarchical attributes based on whether they change with the context-focus specification. The primary attributes are invariant to the selection of context and focus; the secondary attributes are invariant to the focus; and the tertiary attributes define different ways to focus on a graph. This data structure also specifies the computation hierarchy and minimizes the redundant computation and storage. As a toy example, let us consider the rendering of a circle combined with a square and a diamond. As there are two ways to color the square, and three ways to color the diamond, there are a total of six different rendering outcomes of this data object. Each attribute may also require different computation time. When adding a new feature to the application, we define where the feature lies in the hierarchy and reduce the need to re-compute the same data values. As a result, the front-end display can render faster and allow for instantaneous interaction updates.

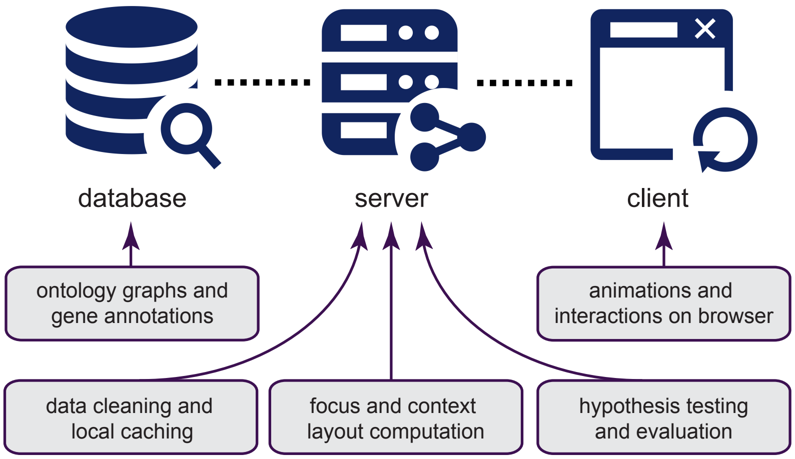

Supplementary Figure 4: System architecture of AEGIS. The system architecture built upon our data hierarchy prioritizes computations required for each attribute the data object to be rendered. In particular, we used a Python Flask server to manage the back-end data communication with the GO database and perform heavy statistical and graph computations on tens of thousands of nodes. All of the user interactions are programmed using javascript packages: jquery.js and D3.js to support light-weight front-end computations on the web-browser. Because existing packages lack the infrastructure to support multiple contexts and focus graphs, we created our own DAG library to define the data hierarchy and perform the core algorithms.

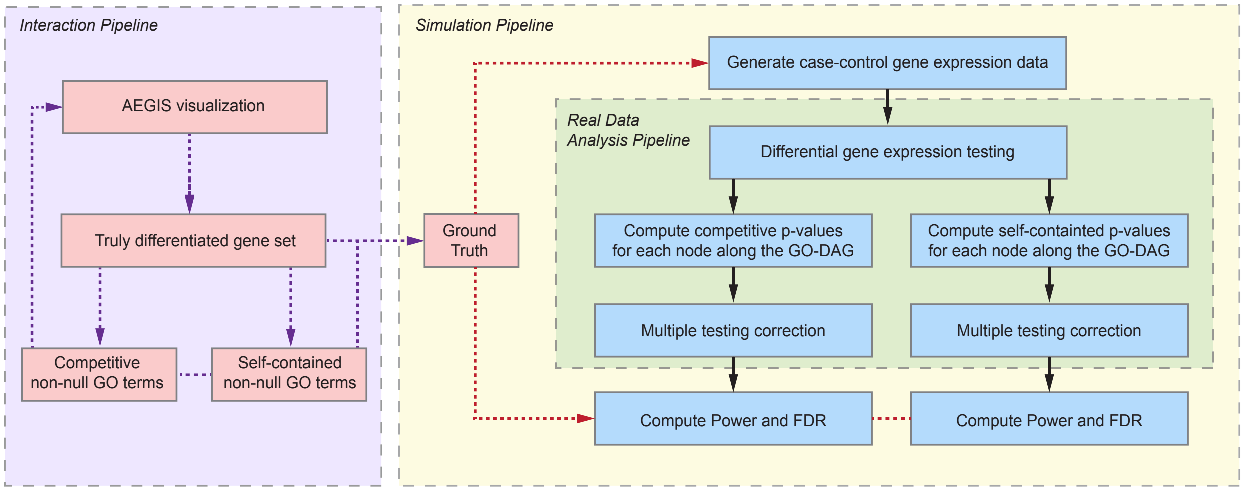

Supplementary Figure 5: AEGIS's power analysis workflow.

The workflow starts with an interaction pipeline where an investigator can use the GO DAG to select specific nodes and draw truly differentially expressed genes from these nodes. The selected genes automatically determine which nodes in the DAG are competitive or self-contained non-nulls. The investigator can make adjustments based on the non-null structures. The ground truth information is then used to generate expression data and evaluate the false discovery rate and power of a typical gene expression analysis pipeline.

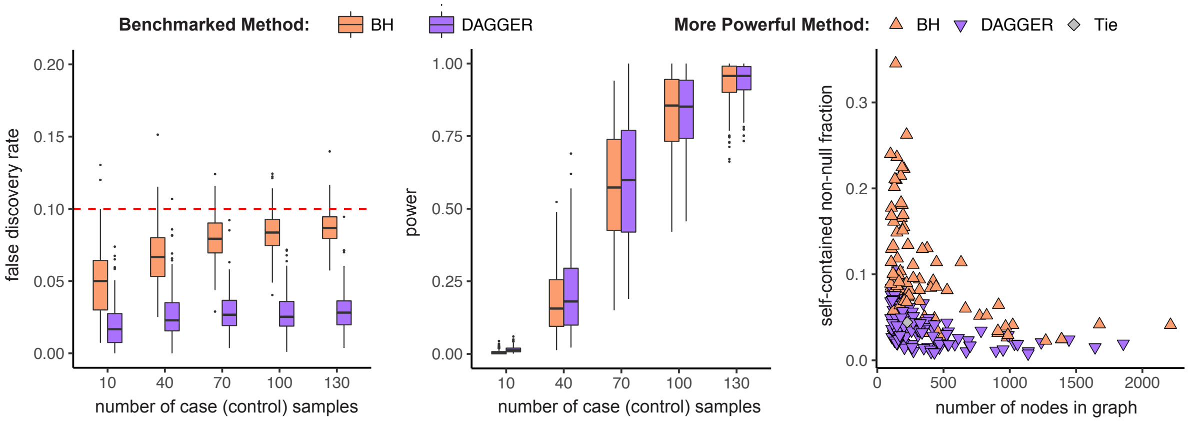

Supplementary Figure 6: Comparison between BH and DAGGER.

The box plots summarizes the false discovery rate (at level 0.1) and power for each of the graphs with 100 repetitions averaged in each regime. The scatter plot indicates the number of nodes and fraction of non-nulls in each of the 200 sub-DAGs. Each plot represents a particular sub-DAG, and the shape and color indicate the method that is more powerful on average. Based on 200 randomly selected sub-DAGs, we observed that both DAGGER and BH have similar power on average. While both control the false discovery rates at their desired levels, DAGGER appears to be more conservative. When we looked at the individual sub-DAGs, we noticed that BH generally tends to outperform DAGGER in regimes when the non-null GO term fraction is high and vice versa. This phenomenon is likely due to the fact that DAGGER sequentially rejects terms by layers: when it stops rejecting on a more general layer, it cannot detect more specific terms lower in the hierarchy.

Supplementary Videos

In addition to the static illustrations in the main paper, we also included video tutorials to illustrate the dynamic visualization and interactions that AEGIS offers. A total of four video tutorials are hosted at `http://aegis.stanford.edu/tutorial/`.

0.1 Introduction to Focus and Context Graphs

This tutorial introduces the concept of focus and context graphs, and how they can render results from existing GO analysis pipelines. It also offers as simple demonstration of how to switch highlighting functions and the focus graph. The data example is based on significant GO terms (within the biological process ontology) identified from a GWAS study[1].

0.2 Interactions to Explore the GO

This tutorial demonstrates how to explore the GO without any data inputs. It also demonstrates how to use search auto-complete features, customize the visualizations, and select functionally related gene sets (used for a single-cell analysis simulation, for example).

0.3 Overview of Graph Layouts

This tutorial gives an overview of the graph layouts, and some of the interactions on the focus graphs implemented in AEGIS. In particular, the buoyant layout is featured and compared with two other layouts: the root-bound and the leaf-bound layouts. The data example is based on enriched GO terms (within the cellular component ontology) from a ChIP-seq study[2].

0.4 Power Analysis Workflow

This tutorial illustrates the interactive workflow to setup the power analysis with AEGIS. It demonstrates how to customize the context graph to determine the terms to be tested. It also demonstrates how to highlight non-null GO terms based on the signal gene set selection within the interface. The demonstration is performed within the full AEGIS interface, which requires local installation.

Supplementary Algorithms

Supplementary Algorithm 1: The constrained GO DAG refinement algorithm

Algorithm: DAG Refinement with Constraints

Input: Full DAG fulldag with (up-propagated) node weights
Param: wmin and wmax, restriction on the output GO term sizes
Output: Refined DAG refinedag

```
refinedag ← {};
foreach node in fulldag do
    if wmin ≤ weight(node) ≤ wmax then
        add(node, refinedag);
foreach node in refinedag do
    edgeupdate(node, refinedag);
queue ← findroots(refinedag);
while queue ≠ ∅ do
    currentcount ← length(queue);
    while currentcount > 0 do
        node ← popleft(queue);
        currentcount ← currentcount −1;
        if node not in refinedag then
            /* do nothing as this node is not in the DAG anymore    */
            continue;
        if weight(node) == maxchildweight(node) then
            /* this node is redundant w.r.t. one of its children    */
            foreach cnode in children(node) do
                parents(cnode) ← parents(cnode) ∪ parents(node);
                parents(cnode) ← parents(cnode) − node;
            foreach pnode in parents(node) do
                children(pnode) ← children(pnode) ∪ children(node);
                children(pnode) ← children(pnode) − node;
            remove(node, refinedag);
        foreach cnode in children(node) do
            pushright(queue, cnode);
```

Supplementary Algorithm 2: The bubble float algorithm

```
Algorithm: Bubble Float
Input: dag, a list of nodes with weight, depth, and parents attributes
Param: cutoff, a threshold that limits the node weight range per level
Output: levelnodes, a list of equal-level node lists.
/* First, each node is initialized to be in its own layer after
   sorting.  The nodes are sorted by first descending order of
   their weights (assumed to be consistent with the topological
   order), and then by ascending order of their depths (defined as
   the longest paths from the root set).                          */
sortednodes ← sort(dag, by = (−weight, depth));
levelnodes ← [ ];
foreach node in sortednodes do
    pushright(levelnodes, [node])
/* Next, we iterate through each layer from the bottom to top.   */
curr ← length(sortednodes) − 1;
while curr > 0 do
    currqueue ← levelnodes [curr];
    next ← curr − 1;
    nextnode ← levelnodes [next][0]; /* next level is always singleton */
    /* For nextnode in the next level, maxchildweight is maximum of
       the weights of all of its children in the current level.   */
    maxchildweight ← 0;
    foreach node in currqueue do
        if nextnode in node.parents then
            maxchildweight ← max{ maxchildweight, node.weight };
    /* The following is the core of the float operation.          */
    while length(currqueue) > 0 do
        /* Do not float any nodes with weight less than the value
           (nextnode − cutoff), and also do not float any nodes that
           have weight less than or equal to maxchildweight.      */
        if currqueue[0].weight < (nextnode − cutoff) then
            break;
        if currqueue[0].weight <= maxchildweight then
            break;
        /* Otherwise, nodes that pass the two criteria are allowed
           to move up to the next level.  (The float operation).  */
        floatnode ← popleft(currqueue);
        pushright(levelnodes[next], floatnode);
    curr ← curr − 1;
levelnodes ← removeemptylevels(levelnodes);
```

Supplementary Algorithm 3: The focus graph node ordering algorithm

Algorithm: Focus Graph Node Ordering

```
Input: DAG dag, which includes a collection of leveled nodes
Param: (Optional) anchors, a set of nodes whose relative nodes cover dag
Output: dag, the graph with computed position attributes
Function HierarchicalReording(dag, relation, reverse):
    maxlevel ← max(getattr(dag, "level"));
    if reverse then
        levellist ← [maxlevel − 1, maxlevel − 2, ..., 1, 0];
    else
        levellist ← [1, 2, ..., maxlevel − 1, maxlevel];
    foreach currentlevel in levellist do
        currentlevelnodes ← getnodes(dag, currentlevel) ;
        foreach currentnode in currentlevelnodes do
            neighbornodes ← getnodes(currentnode, relation);
            neighborpositions ← getattr(neighbornodes, "position");
            setattr(currentnode, "score", mean(neighborpositions));
        rank ← order(getattr(currentlevelnodes, "score"));
        setattr(currentlevelnodes, "position", rank);
    return dag;
if empty(anchors) then
    foreach node in dag do setattr(node, "position", 0);
else
    /* greedily group related anchors and their relative nodes */
    nodegroups ← partition (dag, anchors);
    groupoffset ← 0;
    foreach nodegroup in nodegroups do
        foreach node in nodegroup do
            setattr(node, "position", groupoffset);
        groupoffset ← groupoffset + computewidth(nodegroup);
dag ← HierarchicalReording(dag, "children", reverse = false);
dag ← HierarchicalReording(dag, "parent", reverse = true);
```

Supplementary Note 1: Theoretical Analysis of the Algorithms

1.1 Notation and definitions

Let $G = (\mathcal{V}, \mathcal{E})$ be a directed acyclic graph (DAG), capturing the semantic relationships among N distinct GO terms. As such, we consider the set of GO terms as $\mathcal{V} = \{v_1, ..., v_N\}$, and the set of directed links as $\mathcal{E} = \{(v_i, v_j) : v_j$ is a child term of v_i in the GO$\}$. For each i, let $\mathcal{P}_i$ and $\mathcal{C}_i$ represent the parent set and children set of node v_i respectively. Further, we can generalize the DAG to include a weight set $\mathcal{S}$, where we map each element s_i to be the weight of node v_i.

Definition 1. Suppose that $s_i \geq s_j$ for each $(v_i, v_j) \in \mathcal{E}$ (i.e., every parent node has a weight no less than any of its children nodes), then we say that the weights in set $\mathcal{S}$ are *consistent with the topological ordering* of the DAG.

Specific to an annotated GO DAG, let $\mathcal{G}$ be the total set of genes, and $\mathcal{G}_i \subseteq \mathcal{G}$ be the gene set annotated to the GO term represented by v_i. For our purposes (especially statistical testing and visualization), we use the convention that the gene set annotations are always inherited from a child to its parents, i.e., for each node v_i, if $v_j \in \mathcal{P}_i$, then $\mathcal{G}_i \subseteq \mathcal{G}_j$. Further, we set $s_i = |\mathcal{G}_i|$ (where $|\cdot|$ represents the cardinality of a set), in which case, we also refer to s_i as the *node size* of v_i, and clearly for each v_i, it follows that $s_i \leq s_j$ if $v_j \in \mathcal{P}_i$ by the inheritance property, and the node sizes are always consistent with the topological ordering of the GO DAG. As no cycles exist in the DAG, there must exist a non-empty root set $\mathcal{V}^{\text{root}} = \{v_i \subseteq \mathcal{V} : \mathcal{P}_i = \varnothing\}$ and leaf set $\mathcal{V}^{\text{leaf}} = \{v_i \subseteq \mathcal{V} : \mathcal{C}_i = \varnothing\}$.

Next, we formally define the layout of node levels and some properties.

Definition 2. A (level) *layout* is a function, $f : \mathcal{V} \to \mathbb{Z}$ that assigns each node an integer.

Definition 3. A layout f satisfies the *topological constraint* if $f(v_i) < f(v_j)$ whenever $(v_i, v_j) \in \mathcal{E}$. That is, the parent node is always mapped to a level less than its child nodes.

Definition 4. A layout f satisfies the *descending weight ordering constraint* (or *descending node size constraint* for the GO) if $f(v_i) \geq f(v_j)$ whenever $s_i \leq s_j$ for all pairs $v_i, v_j \in \mathcal{V}$.

Definition 5. A layout is a *root-bound layout* if for each $v \in \mathcal{V}$, the level $f(v)$ is the longest distance from to the root set $\mathcal{V}^{\text{root}}$; a layout is a *leaf-bound layout* if for each $v \in \mathcal{V}$, the level $f(v)$ is the longest distance from to the leaf set $\mathcal{V}^{\text{leaf}}$.

Definition 6. The buoyant layout is any layout f that satisfies both the topological constraint and the descending weight ordering constraint.

1.2 Necessary and sufficient conditions for the buoyant layout

It is easy to show that the root-bound layout satisfies the topological constraint; after negating the levels the image of the level mapping of the leaf-bound layout, one can also similarly show that it satisfies the topological constraint. The existence of these two layouts follow from the definition of DAGs, and does not rely on node weights. However, when the node weights are included, we need to consider both the DAG structure and the weights simultaneously. Here, our goal is to justify the buoyant layout for this purpose.

Without loss of generality, we assume that $f(v) = 0$ for all $v \in \mathcal{V}^{\text{root}}$, i.e., the root nodes are placed at the highest level 0, and their descendant nodes are placed below this level. Note that the topological constraint highlights the topological ordering of the DAG because for any two nodes that have an ordering, the parent node is always placed at a higher level than the child node. The descending node weight constraint applies to all pairs of nodes in the DAG whether or not there is a directed path between them. Below, we show a strong mathematical guarantee of buoyant layout.

Theorem 1. For any weighted DAG, there exists a buoyant layout if and only if the weights are consistent with the topological ordering of the DAG.

Proof. First, we prove the existence of a buoyant layout requires weight consistency by contradiction. Suppose that there exists a buoyant layout where the weights are not consistent with the topological ordering. That is, there exists $(v_i, v_j) \in \mathcal{E}$ such that $s_i < s_j$. Then the descending weight ordering constraint implies that $f(v_i) \geq f(v_j)$, contradicting the topological constraint. Second, we prove the other direction by constructing a layout that satisfies both constraints assuming weight consistency. We define g_i to be the longest path from v_i to nodes in the root set $\mathcal{V}^{\text{root}}$ (which is non-empty). For each node v_i, consider an ordered tuple $(-s_i, g_i)$, and let f be the strict lexicographic order of the nodes based on this tuple, i.e.,

$$f(v_i) < f(v_j) \text{ if and only if } s_i > s_j \text{ or } (s_i = s_j \text{ and } g_i < g_j).$$

Here, ties are broken arbitrarily such that no two nodes share a layer, i.e., $f(v_i) \neq f(v_j)$ for any pair $v_i, v_j \in \mathcal{V}$. By considering the first comparisons on the node weights, clearly the descending weight ordering constraint is satisfied. To show the topological ordering constraint holds, we only need to consider the pairs of comparable nodes $(v_i, v_j) \in \mathcal{E}$. By the definition of weight consistency, it also must hold that $s_i \geq s_j$. For such pairs, we always have that $g_i < g_j$ because v_i is a parent node and must be closer to the root (or else there would be a cycle). Thus, we have that $f(v_i) < f(v_j)$ for all $(v_i, v_j) \in \mathcal{E}$, which completes the proof. □

1.3 Analysis of the bubble float algorithm

At each step of the float operation, there is a singleton in the next (higher) level to be considered, and all of its out links are visited exactly once to ensure that none of its descendants move to its level. Moreover, the number of float operations is at most the number of nodes, where the search for which nodes to float by weight can be logarithmic or constant with appropriate data structures. Taken together, the worst case runtime is $\mathcal{O}(|\mathcal{E}|+|\mathcal{V}|^2)$ with space requirement $\mathcal{O}(|\mathcal{E}|+|\mathcal{V}|)$, which can also just be considered as order $\mathcal{O}(|\mathcal{E}|)$ since $|\mathcal{E}|$ can be quadratic in $|\mathcal{V}|$ in the worst case. In practice, the runtime for the full GO scales almost linearly with the number of nodes as the links are relatively sparse. Next, we prove the correctness of the algorithm.

Theorem 2. The bubble float algorithm generates a buoyant layout which satisfies both the topological constraint and the descending weight ordering constraint.

Proof. The bubble float algorithm is an iterative algorithm which terminates when $|\mathcal{V}| - 1$ float operations are made. Each float operation decides which nodes to move from the current level to the next level. During the initialization, the layout is a buoyant layout as we have shown in the proof of Theorem 1. Therefore, to show that it produces a buoyant layout, it is sufficient to (inductively) show that each float operation preserves both the topological constraint and the descending weight ordering constraint. At the i-th iteration, suppose the current configuration satisfies the buoyant layout. At this iteration, the float operation only changes the $(|\mathcal{V}| - i)$-th and the $(|\mathcal{V}| - i - 1)$-th level. The higher level has a singleton node, and the lower level can have multiple nodes. By our construction, the nodes that move from the lower level to the higher level cannot be a descendant of the node on the higher level, and therefore, preserving the topological constraint. Further, if k nodes float, the nodes must be the k largest nodes (none of which can be greater than the singleton node on the higher level) such that they will all be greater than the remaining nodes left in the lower level. Hence, the descending weight ordering constraint is also preserved. Taken together, we have shown that the bubble float algorithm outputs a buoyant layout. □

1.4 Analysis of the graph refinement algorithm

Similar DAG reduction approaches have been used by recent works[3,4], but we could not find a full algorithmic description or proof. For completeness, we provide the analysis here.

It is easy to see that the worst-case complexity of the algorithm linear in the number of links (hence at most quadratic in the number of nodes) because each link is visited at most once. To prove the correctness of the algorithm, it is convenient to introduce the following definition of *node annotation redundancy*.

Definition 7. A node v_i is (annotation) redundant if any of its children has the same annotation,

i.e., there exists $v_j \in \mathcal{C}_i$ with $\mathcal{G}_i = \mathcal{G}_j$. By annotation inheritance, it is equivalent to the nodes having the same weight: $s_i = s_j$.

Here we show that DAG refinement removes all node annotation redundancy on the weight-constrained DAG, after observing the minimum and maximum weight requirements.

Theorem 3. The output of the DAG refinement, $\bar{G} = (\bar{\mathcal{V}}, \bar{\mathcal{E}})$ has no node annotation redundancy.

Proof. The algorithm relies on a priority queue equivalent to a breadth-first-search routine, which visits each node based on the link relationships and terminates once all nodes are reached. Each time a node is visited, it is removed from the DAG if it is redundant w.r.t. any of its children. Suppose a node v is removed: then all its children will be re-connected to all its parents. To avoid the degeneracy of having empty nodes in the DAG, we can prune them from the leaves prior to DAG refinement. By the inheritance of gene sets, these empty nodes must start from the leaves. Otherwise, it is also feasible to not prune the empty nodes, as the related empty leaves will be kept. What remains is to show (by contradiction) that after re-connection, the parent nodes of v cannot be redundant with respect to the new children. Suppose that for v, one of its parent node u is redundant w.r.t. one of its child node w. Then the gene sets in nodes u, v, w must all be the same by the inheritance of gene sets. In fact, the parent node u is redundant w.r.t. to v, and must be visited before v (due to the construction of the priory queue) and removed. Hence, we have a contradiction. Taken together, it is guaranteed that every redundant node is removed by the DAG refinement algorithm. The consequence of the algorithm is that for every node removed, there must a non-redundant descendant (and not necessary a direct child) of itself that remains in the refined DAG. □

Supplementary Note 2: Framework for testing for enrichment

2.1 Notation Summary

For statistical testing on the GO DAG, we adapt notations in 1 and introduce some additional notations. Because not all terms may be tested in the GO DAG, we use $\mathcal{M} \subseteq \mathcal{V}$ to denote the set of nodes that are tested, and one can use $\mathcal{M}$ to construct a corresponding DAG that preserves node relations based on the original DAG. Next, we let $\mathcal{A}_1, \mathcal{A}_0 \subseteq \mathcal{G}$ denote the truly differentiated gene set (or the signal genes) and the other (non-signal) genes respectively; the choice of $\mathcal{A}_0, \mathcal{A}_1$ leads to the set of null and non-null GO terms, denoted as $\mathcal{M}_0, \mathcal{M}_1 \subseteq \mathcal{M}$, with $\mathcal{M}_1 = \mathcal{M} \setminus \mathcal{M}_0$ (the set difference). To distinguish self-contained and competitive hypotheses, we use superscripts "self" and "comp" to distinguish the corresponding hypothesis type.

Symbol	Meaning
$\lvert \cdot \rvert$	the cardinality of a set
$\setminus$	the set difference
$\mathcal{G}$	index set of all genes measured, i.e., $\{1, 2, ..., \lvert\mathcal{G}\rvert\}$
$\mathcal{M}$	index set of GO terms to be tested, i.e., $\{1, 2, ..., \lvert\mathcal{M}\rvert\}$
$\mathcal{G}_i$	subset of genes annotated with the i-th GO term ($\mathcal{G}_i \subseteq \mathcal{G}$)
$\mathcal{A}_1$	set of truly differentially expressed genes, or non-null genes ($\mathcal{A}_1 \subseteq \mathcal{G}$)
$\mathcal{A}_0$	set of true null genes, i.e., $\mathcal{G} \setminus \mathcal{A}_1$
$H_{0,i}^{\text{self}}$, $H_{0,i}^{\text{comp}}$	self-contained/ competitive null hypothesis for the i-th GO term
$H_{1,i}^{\text{self}}$, $H_{1,i}^{\text{comp}}$	alternative self-contained/ competitive hypothesis for the i-th GO term
$\mathcal{M}_0^{\text{self}}$, $\mathcal{M}_0^{\text{comp}}$	the set of self-contained/ competitive null hypotheses ($\mathcal{M}_0^* \subseteq \mathcal{M}$)
$\mathcal{M}_1^{\text{self}}$, $\mathcal{M}_1^{\text{comp}}$	the set of self-contained/ competitive non-null hypotheses ($\mathcal{M}_1^* \subseteq \mathcal{M}$)
$n_{\text{control}}, n_{\text{case}}$	number of control/case samples
β_{effect}	(non-zero) effect size for each non-null gene in $\mathcal{A}_1$
p_j^{gene}	the p-value of gene j from differential testing between case/control
$\mathcal{D}$	set of significantly differentially expressed genes in the data ($\mathcal{D} \subseteq \mathcal{G}$)
p_i^{simes}, p_i^{hyper}	the p-value of node i based on the Simes'/ hypergeometric test
$\mathcal{R}^*$	set of rejected GO terms after multiplicity correction; $\mathcal{R}^* \subseteq \mathcal{M}$

2.2 Self-contained and competitive hypotheses

Here, we formally define two types of nulls (and their counterpart non-nulls): the *self-contained* null set $\mathcal{M}_0^{\text{self}}$ (and non-null $\mathcal{M}_1^{\text{self}}$), and the competitive null set $\mathcal{M}_0^{\text{comp}}$ (and non-null $\mathcal{M}_1^{\text{comp}}$)[5]. Recall that $\mathcal{G}_i$ represents the set of genes annotated to the i-th GO term. Then, we say that the i-th GO term is a self-contained null if no genes in $\mathcal{G}_i$ are differentially expressed. On the other hand, the i-th GO term is a competitive null if the genes in $\mathcal{G}_i$ are at most as often differentially expressed as the genes in $\mathcal{G} \setminus \mathcal{G}_i$. In other words, each node has a ground-truth contingency table:

	non-null genes	null genes	total
genes annotated with GO term i	$\lvert\mathcal{A}_1 \cap \mathcal{G}_i\rvert$	$\lvert\mathcal{G}_i \setminus \mathcal{A}_1\rvert$	$\lvert\mathcal{G}_i\rvert$
genes not annotated with GO term i	$\lvert\mathcal{A}_1 \setminus \mathcal{G}_i\rvert$	$\lvert(\mathcal{G} \setminus \mathcal{G}_i) \setminus \mathcal{A}_1\rvert$	$\lvert\mathcal{G} \setminus \mathcal{G}_i\rvert$
total	$\lvert\mathcal{A}_1\rvert$	$\lvert\mathcal{G} \setminus \mathcal{A}_1\rvert$	$\lvert\mathcal{G}\rvert$

Accordingly, the corresponding self-contained and competitive null hypotheses are defined as:

$$H_{0,i}^{\text{self}} \,:\, \mathcal{A}_1 \cap \mathcal{G}_i = \varnothing, \quad H_{0,i}^{\text{comp}} : |\mathcal{A}_1 \cap \mathcal{G}_i|/|\mathcal{A}_1| \le |\mathcal{G}_i|/|\mathcal{G}|;$$

or, in terms of the null sets, we have

$$\mathcal{M}_0^{\text{self}} = \{i \in \mathcal{M} :\, \mathcal{A}_1 \cap \mathcal{G}_i = \varnothing\},\ \mathcal{M}_0^{\text{comp}} = \{i \in \mathcal{M} :\, |\mathcal{A}_1 \cap \mathcal{G}_i|/|\mathcal{A}_1| \le |\mathcal{G}_i|/|\mathcal{G}|\}.$$

Note that $|\mathcal{A}_1 \cap \mathcal{G}_i|/|\mathcal{A}_1| \le |\mathcal{G}_i|/|\mathcal{G}|$ is also equivalent to $|\mathcal{A}_1 \cap \mathcal{G}_i|/|\mathcal{A}_1| \le |\mathcal{G}_i \setminus \mathcal{A}_1|/|\mathcal{G} \setminus \mathcal{A}_1|$.

Importantly, the ground truth information $\mathcal{A}_1$, $\mathcal{M}_0^{\text{self}}$, $\mathcal{M}_0^{\text{comp}}$ are defined *a priori*, i.e., before generating the gene expression data, and are not observable by any testing methods.

2.3 Data Generation

Given a set of ground-truth non-null genes $\mathcal{A}_1$, effect size β_{effect} and sample sizes n_{control}, n_{case} as parameters, we generate $|\mathcal{G}|$-dimensional gene expression matrices for the control samples and case samples. By default, the n_{control} controls are sampled from $\mathcal{N}(\mathbf{0}, \mathbf{I})$, a multivariate normal distribution with a length-$|\mathcal{G}|$ zero mean vector and a covariance matrix equal to the identity matrix; and the n_{case} cases are similarly sampled from $\mathcal{N}(\mu, \mathbf{I})$, where each element of μ is given by

$$\mu_j = \begin{cases} \beta_{\text{effect}}, & \text{if } j \in \mathcal{A}_1, \\ 0, & \text{if } j \in \mathcal{A}_0. \end{cases}$$

In the default model, we assume the gene measurements are uncorrelated, but this assumption can be easily relaxed given the knowledge of platform-specific noise patterns.

Next, we perform a standard two-sample t-test between case and control samples for each gene, resulting in a set of differential gene p-values: $\{p_j^{\text{gene}}\}_{j=1}^{|\mathcal{G}|}$, These are typical p-values that one would compute in a differential gene expression analysis: based on different assumptions for the generating distribution, the statistical tests can be chosen differently.

2.4 Self-contained and Competitive testing procedures

To compute the node p-value for each GO-term given the computed gene p-values, we consider two different approaches: 1) the Simes' test for the self-contained null, and 2) the hypergeometric test for the competitive null.

The Simes's test[6] is a global testing procedure that tests if all hypotheses in a group are null. For node i, Simes' computes a p-value based on the ascending gene p-values from the set $\{p_j^{\text{gene}} : j \in \mathcal{G}_i\}$, denoted as $p_{(1)}^{\text{gene}}, ...p_{(|\mathcal{G}_i|)}^{\text{gene}}$:

$$p_i^{\text{simes}} = \min_{j=1,\ldots,|\mathcal{G}_i|} \left\{ p_{(j)} \frac{n}{j} \right\}.$$

Under the global null that all the genes are null in the set $\mathcal{G}_i$ (or $\mathcal{A}_1 \cap \mathcal{G}_i = \varnothing$), it is easy to show that the p_i^{simes} is uniformly distributed as long as the null gene p-values are uniformly distributed.

The hypergeometric test is a common test[7,8] used to test over-representation of the gene set annotated to a term. It starts with a gene set of significantly differentially expressed genes suggested by the data, which we denote as $\mathcal{D}$. Following typical workflows, we construct $\mathcal{D} = \{j \in \mathcal{G} : p_j^{\text{gene}} \leq \alpha^{\text{gene}}\}$, where α^{gene} is the gene-wide significance threshold (accounting for multiple testing adjustment). Then, the test is based on the following *data* contingency table: The

	significant genes	non-significant genes	total
genes annotated with GO term i	$\lvert\mathcal{D} \cap \mathcal{G}_i\rvert$	$\lvert\mathcal{G}_i \setminus \mathcal{D}\rvert$	$\lvert\mathcal{G}_i\rvert$
genes not annotated with GO term i	$\lvert\mathcal{D} \setminus \mathcal{G}_i\rvert$	$\lvert(\mathcal{G} \setminus \mathcal{G}_i) \setminus \mathcal{D}\rvert$	$\lvert\mathcal{G} \setminus \mathcal{G}_i\rvert$
total	$\lvert\mathcal{D}\rvert$	$\lvert\mathcal{G} \setminus \mathcal{D}\rvert$	$\lvert\mathcal{G}\rvert$

p-value of the hypergeometric test is the following probability:

$$p_i^{\text{hyper}} = \Pr(X \geq |\mathcal{D} \cap \mathcal{G}_i| \; ; \; n = |\mathcal{D}|, N = |\mathcal{G}|, K = |\mathcal{G}_i|),$$

where X can be interpreted as the number of white balls among n balls randomly drawn from an urn with a total of $|\mathcal{G}_i|$ white balls and $|\mathcal{G}| - |\mathcal{G}_i|$ black balls.

2.5 Multiple Testing Correction

Finally, the significant GO-terms for each type of test can be selected by a multiple testing procedure using the set of Simes' p-values $\{p_i^{\text{simes}}\}_{i=1}^{|\mathcal{M}|}$, or the hypergeometric p-values $\{p_i^{\text{hyper}}\}_{i=1}^{|\mathcal{M}|}$ respectively. In our case, we used the Benjamini-Hochberg (BH) procedure to aim to control the false discovery rate (FDR) at level q. Other options, such as the Bonforreni correction, are also available. For the benchmark example, we compared two multiplicity correction methods: DAGGER[9], a sequential testing algorithm which rejects hypothesis hierarchically, with BH which rejects hypotheses simultaneously but is unaware of the structure (Supplementary Figure 6).

Supplementary Note 3: Case Studies

3.1 Visualization of enriched terms in the GWAS example

The 27 enriched GO terms for the GWAS example were extracted from Supplementary Table 8 of the original study on hair color variation[1]. Because some of GO term identifiers were based on an older version of the GO, we manually translated the terms to the current version. For convenience, these terms are also part of an example provided in the web interface of AEGIS. Given the list of 27 term identifiers as inputs, we created two visual displays for illustration with AEGIS. For both cases, we used the "biological process" ontology with human gene annotations.

First, we rendered the static visualization in Main Figure 1 by setting the 27 terms as the input query data in the full version of AEGIS. We kept the full context graph under the root anchor of "biological process" (GO: 0008150), and selected all the 27 terms as leaf anchors of the focus graph. In the example, we used the root-bound layout option instead of the default buoyant layout option. The node highlight option of "focus anchor relatives" was selected to indicate the total number of ancestors and descendants among the full ontology and pinpoint the 27 terms. After downloading the raw image, we later added the text annotations and adjust the legends.

Second, we created the interactive visualization in Supplementary Video 1, by setting the 27 terms as the input query data in the light version of AEGIS (also part of our web showcase). The context graph was constructed based on the 27 terms as waypoint anchors, so that only terms directly related to the queries were kept. To show how to interact with fewer terms at a time, we limited the number of query terms that could be used as leaf anchors for the focus graph. In the video demonstration, we used the default buoyant layout and adjusted some of the focus graph parameters among the existing options.

3.2 Visualization of enriched terms in the ChIP-seq study

To obtain the significant GO terms in the ChIP-seq study[2], we used the demonstrative example with the GREAT[3] web interface (version 3.0.0). The interface identified nine enriched terms under the "cellular component" ontology. Accordingly, we used the same ontology and human gene annotations as the options for AEGIS. For convenience, these terms are also part of an example provided in its web interface. Within the interface, the context graph was constructed based on the nine terms as waypoint anchors, resulting 94 nodes in the context. The illustration in Supplementary Video 2 used all 9 terms as leaf anchors for the focus graph (by default), and the function to switch between root-bound, leaf-bound and buoyant layouts. To provide the example in Main Figure 2, we used only two of the focus anchors: "ruffle" (GO:0001726) and "actin cytoskele-

ton" (GO: 0014629). The colors, legends, and annotations were manually adjusted for illustrative purpose in the figure (and not part of the common node highlight options in the software).

3.3 Simulation of single-cell expression distinguished by cell cycle signatures

The goal of this analysis is to improve existing benchmarks for scRNA-seq analysis, where modeling realistic data is currently an active area of research[10]. This has practical implications for real data analysis: instead of solely answering "which cells are grouped together", it is also key to explain "how are the cell groups functionally different".

The steps to retrieve the gene sets are as part of Supplementary Video 3. At the output of AEGIS, we randomly selected 10 genes respectively each from three descendants of the "cell cycle process" (GO:0022402): "G1/S transition of mitotic cell cycle" (GO:0000082), "G2/M transition of mitotic cell cycle" (GO:0000086), and "mitotic cell cycle arrest" (GO:0071850). We refer to these 30 genes as the signature genes.

As most scRNA-seq pipelines filter out genes with low variability as pre-processing step, we randomly selected 2060 genes (including the signature genes) to be our variable genes. This scale is common for scRNA-seq studies[11], especially those captured with droplet technologies[12]. In the example, we used 120, 150, 100, 300 cells for G1/S transition, G2/M transition, cell cycle arrest, and control respectively. Each cell type is supposed to have enriched expression of its 10 signature genes; the control cell type is the baseline and does not have enrichment of any of the signature genes. To model the counts in the matrix, we chose a zero-inflated negative binomial model[13]. Under this model, signature and non-signature genes entail different negative binomial parameters. The non-signature follows a negative binomial with parameters $n = 30$, $p = 0.85$, where n is interpreted as the number of failures and p is the probability of success. To capture technical effects, we also added stochastic drop-out events: randomly setting 80% of the values to zero, a common characteristic of scRNA-seq data. For the signature genes, we set gene-specific parameters for p based on a normal distribution with mean 0.8 and variance 0.1. Only 50% of the genes for the population-specific signature gene expression were set to zero. For efficiency, we generated the expression of the signature genes via a block diagonal matrix. The setup resulted in a gene expression matrix with a total of 670 cells and 2060 genes.

The secondary analysis includes three visual components: 1) heatmap view of the expression, 2) projection of the cells in the PC space and 3) the embedding of the cells with t-SNE. These are among the most common techniques for exploratory analysis, even though numerous variants exist. For the heatmap, we organized the genes and cell types according to cell types, and included 10 random non-signal genes for the controlled population. For better contrast, we transformed the scale to log-scale z-score: the data matrix was log-transformed, and each gene was standardized.

PCA is a critical step in dimension reduction. It is also one of the most interpretable unsupervised learning approaches that we can leverage for insightful representation of the data. We also projected the signal genes in the space spanned by the first two PCs, resulting in a biplot. We adjusted the scale of the gene vectors in the PC space by the power of 0.4 and compensated for the scaling among the projected data points. Finally, the t-SNE projection was based on the top 10 PCs in the data, as the remaining PCs each explained little of the variance of the data.

The complete step-by-step guide for our workflow can be found as part of our notebooks at: `http://aegis.stanford.edu/notebooks/aegis_single_cell_example.html`.

3.4 Power analysis for the heart development study

This case study was inspired by a study[14] of tissue-specific expression from the GTEx project[15]. The study identified over 50 tissue-enriched processes via the GO. Among the examples, we found that the term "heart development" (GO:0007507) was uniquely associated with the heart tissue. Interestingly, AEGIS revealed that "heart development", along with development processes in other tissues are descendants of "animal organ development" (GO:0048513). Then, we designed a power analysis to guide a hypothetical confirmatory study with limited sample; the goal of follow-up study was to detect heart-specific developmental processes among all known organ development processes in different tissues.

During the interaction as demonstrated in Supplementary Video 4, we selected the context graph rooted at "animal organ development" (GO:0048513), which has 26 immediate children terms, including "heart development" (GO:0007507), and 105 total descendant terms. The nodes in the context graph are individual GO term hypotheses to be tested. For the ground truth parameters, we selected all 15 genes (each with effect size 0.5) that have been previously annotated to "adult heart development" (GO:0007512). This term is also the only child of "heart development". The signal genes suggested 16 self-contained, or 13 competitive non-null GO terms, whose non-null covers generated the binder plot in Figure 4 (also available in the full version of AEGIS). Note that the 16 self-contained nodes are the non-null cover of itself, and that of the 13 competitive nodes - so we indicated the 3 null nodes in the cover by coloring the node and text in gray. We highlighted "adult heart development" with the blue node borders, and also curved links to indicate its relationship with "heart development" to "animal organ development". The other non-null nodes shown in the binder plot are not semantically related to "adult heart development" but are selected as non-nulls because they share some of the genes in "adult heart development".

The terms in the binder plot are linearly ordered by descending node sizes, and the different level groups are determined by the buoyant layout, such that both the topological constraint and descending node size constraints are preserved. To generate the graph with the web-interface, we forced the focus graph to take all the 16 self-competitive non-nulls as leaf anchors.

The simulation consisted of 10 regimes (with number of samples ranging from 10 to 100 with equal case and control samples), and each regime was repeated 100 times during the simulation. The hypergeometric test was used to generate node p-values under the competitive hypotheses, and the Simes' test for

the self-contained hypotheses. For both simulations, BH was used for multiplicity adjustment. As part of the web-interface of AEGIS (simulation result visualizer), we augmented the binder plots with heatmaps to highlight the fraction of times (among the 100 repetitions) a particular node in the non-null cover is rejected for in each of the 10 regimes, or the node-specific power. To characterize the overall power and FDR in the multiple testing problem involving the 106 hypotheses, we used box plots to summarizes the false discovery proportion (at level 0.1) and power for each of the repetitions across each regime. The plots were generated using standard outputs from AEGIS and plotting tools in Python, which we include as part of our notebook tutorials.

3.5 Comparison between DAGGER and BH

The workflow for comparing DAGGER and BH is based on the power analysis workflow described above, with the difference that the context graph used for testing is not fixed. We drew 200 random sub-DAGs from the GO with root nodes that ranged from size 500 to 10000 genes. It was also ensured that each DAG had at least 100 terms. Then we placed up to 5 signal genes randomly selected from a leaf node of each DAG. Each sub-DAG was associated with a particular set of signal genes. The term p-values were computed based on the Simes' test and evaluated on self-contained non-null hypotheses. For each graph, we used mainly parameters of the simulation setup for AEGIS, but we only considered 5 sample-size regimes (each with 100 repetitions varying the random noise in the the data matrix). We used the box plots to summarize the FDR (at level 0.1) and power for each of the graphs with 100 repetitions averaged in each regime. To generate the scatter plot, we compared the 200 sub-DAG where BH had higher power on average than DAGGER, and vice versa; then, we include the two graph characteristics for the scatter plot: the number of nodes and the fraction of non-nulls.

For completeness, we provided step-by-step guide notebooks with two parts for this comparison. The first part explains how to use the AEGIS software API to conduct benchmarks with random graphs. The second part takes the data and ground-truth outputs from the first part to evaluate specific methods. Although AEGIS is implemented in Python, we specifically used the R programming language for the second part to showcase that the data outputs are universally applicable to different programming environments. The two parts can be found at:
`http://aegis.stanford.edu/notebooks/aegis_benchmark_part1.html`
`http://aegis.stanford.edu/notebooks/aegis_benchmark_part2.html`.

Supplementary References

1. Hysi, P. G. *et al.* Genome-wide association meta-analysis of individuals of European ancestry identifies new loci explaining a substantial fraction of hair color variation and heritability. *Nature Genetics* **50**, 652–656 (2018).

2. Valouev, A. *et al.* Genome-wide analysis of transcription factor binding sites based on ChIP-Seq data. *Nature Methods* **5**, 829 (2008).

3. McLean, C. Y. *et al.* GREAT improves functional interpretation of cis-regulatory regions. *Nature Biotechnology* **28**, 495 (2010).

4. Ma, J. *et al.* Using deep learning to model the hierarchical structure and function of a cell. *Nature Methods* **15**, 290 (2018).

5. Goeman, J. J. & Bühlmann, P. Analyzing gene expression data in terms of gene sets: methodological issues. *Bioinformatics* **23**, 980–987 (2007).

6. Benjamini, Y. & Heller, R. Screening for partial conjunction hypotheses. *Biometrics* **64**, 1215–1222 (2008).

7. Huang, D. W., Sherman, B. T. & Lempicki, R. A. Systematic and integrative analysis of large gene lists using DAVID bioinformatics resources. *Nature Protocols* **4**, 44 (2008).

8. Carbon, S. *et al.* AmiGO: online access to ontology and annotation data. *Bioinformatics* **25**, 288–289 (2009).

9. Ramdas, A., Chen, J., Wainwright, M. J. & Jordan, M. I. DAGGER: A sequential algorithm for FDR control on DAGs. *arXiv preprint arXiv:1709.10250* (2017).

10. Huang, M. *et al.* SAVER: gene expression recovery for single-cell RNA sequencing. *Nature Methods* **15**, 539–542 (2018).

11. Butler, A., Hoffman, P., Smibert, P., Papalexi, E. & Satija, R. Integrating single-cell transcriptomic data across different conditions, technologies, and species. *Nature Biotechnology* **36**, 411 (2018).

12. Zheng, G. X. Y. *et al.* Massively parallel digital transcriptional profiling of single cells. *Nature Communications* **8**, 14049 (2017).

13. Risso, D., Perraudeau, F., Gribkova, S., Dudoit, S. & Vert, J.-P. A general and flexible method for signal extraction from single-cell RNA-seq data. *Nature Communications* **9**, 284 (2018).

14. Feiglin, A., Allen, B. K., Kohane, I. S. & Kong, S. W. Comprehensive Analysis of Tissue-wide Gene Expression and Phenotype Data Reveals Tissues Affected in Rare Genetic Disorders. *Cell Systems* **5**, 140–148.e2 (2017).

15. Consortium, G. The Genotype-Tissue Expression (GTEx) pilot analysis: Multitissue gene regulation in humans. *Science* **348**, 648–660 (2015).
